# VAV1-Cre mediated hematopoietic deletion of CBL and CBL-B leads to JMML-like aggressive early-neonatal myeloproliferative disease

**DOI:** 10.18632/oncotarget.10638

**Published:** 2016-07-16

**Authors:** Wei An, Bhopal C. Mohapatra, Neha Zutshi, Timothy A. Bielecki, Benjamin T. Goez, Haitao Luan, Fany Iseka, Insha Mushtaq, Matthew D. Storck, Vimla Band, Hamid Band

**Affiliations:** ^1^ Eppley Institute for Research in Cancer and Allied Diseases, University of Nebraska Medical Center, Omaha, NE 68198, USA; ^2^ Departments of Genetics, Cell Biology and Anatomy, University of Nebraska Medical Center, Omaha, NE 68198, USA; ^3^ Departments of Biochemistry and Molecular Biology, University of Nebraska Medical Center, Omaha, NE 68198, USA; ^4^ Departments of Pathology and Microbiology, College of Medicine, University of Nebraska Medical Center, Omaha, NE 68198, USA; ^5^ Departments of Fred and Pamela Buffet Cancer Center, University of Nebraska Medical Center, Omaha, NE 68198, USA

**Keywords:** CBL, ubiquitin ligase, HSC, neonatal hematopoiesis, JMML

## Abstract

CBL and CBL-B ubiquitin ligases play key roles in hematopoietic stem cell homeostasis and their aberrations are linked to leukemogenesis. Mutations of CBL, often genetically-inherited, are particularly common in Juvenile Myelomonocytic Leukemia (JMML), a disease that manifests early in children. JMML is fatal unless corrected by bone marrow transplant, which is effective in only half of the recipients, stressing the need for animal models that recapitulate the key clinical features of this disease. However, mouse models established so far only develop hematological malignancy in adult animals. Here, using VAV1-Cre-induced conditional CBL/CBL-B double knockout (DKO) in mice, we established an animal model that exhibits a neonatal myeloproliferative disease (MPD). VAV1-Cre induced DKO mice developed a strong hematological phenotype at postnatal day 10, including severe leukocytosis and hepatomegaly, bone marrow cell hypersensitivity to cytokines including GM-CSF, and rapidly-progressive disease and invariable lethality. Interestingly, leukemic stem cells were most highly enriched in neonatal liver rather than bone marrow, which, along with the spleen and thymus, were hypo-cellular. Nonetheless, transplantation assays showed that both DKO bone marrow and liver cells can initiate leukemic disease in the recipient mice with seeding of both spleen and bone marrow. Together, our results support the usefulness of the new hematopoietic-specific CBL/CBL-B double KO animal model to study JMML-related pathogenesis and to further understand the function of CBL family proteins in regulating fetal and neonatal hematopoiesis. To our knowledge, this is the first mouse model that exhibits neonatal MPD in infancy, by day 10 of postnatal life.

## INTRODUCTION

Members of the CBL-family of E3 ubiquitin ligases target activated protein tyrosine kinases (PTKs) as well as components of their immediate signaling machinery for ubiquitination and degradation to provide an evolutionarily-conserved mechanism of negative regulation of PTK-mediated cellular activation [1, 2]. Of the three family members (CBL, CBL-B and CBL-C), CBL and CBL-B are structurally more related, with C-terminal domains/motifs absent in CBL-C, and are the only members expressed in hematopoietic cell lineages [2]. The E3 function of CBL proteins is specified by a highly conserved RING finger (RF) domain and a linker helix region (LHR) that connects the RING finger to an N-terminal tyrosine kinase-binding domain (TKB). The RF domain and LHR together provide an optimal platform for ubiquitin-conjugating enzyme (E2) recruitment and phosphorylation of a conserved tyrosine (Y371 in human CBL), which mediates enzymatic activation of CBL proteins and positions them closer to their PTK targets [1, 3]. Over the past few years, clinical studies have identified mutations of CBL, and rarely CBL-B, in a subset of patients with chronic myeloid leukemia, particularly those within the myeloid dysplastic syndrome/myeloproliferative neoplasm (MDS/MPN) group; the incidence is particularly high among patients with juvenile myelomonocytic leukemia (JMML), chronic myelomonocytic leukemia (CMML) and atypical chronic myeloid leukemia (aCML) [3–9]. Of the leukemias associated with mutations of CBL, JMML presents as an aggressive disease and is typically fatal unless treated with bone marrow transplant, which produces stable remission at 5 years in about half of the patients [10]. Thus, a better understanding of JMML is needed to expand therapeutic choices. Animal models that recapitulate the early postnatal leukemic progression in JMML are therefore highly desirable.

Notably, most JMML patients inherit a heterozygous mutation in CBL or one of a small set of other genes (such as NF1, N-Ras, PTPN11) that regulate RAS signaling, constituting one form of RASopathies [5, 11], with the disease progression invariably associated with a somatic duplication of the mutant allele and concurrent deletion of the wildtype allele in hematopoietic stem cells, referred to as acquired uniparental disomy [7]. A number of mouse models have been engineered to recapitulate features of JMML and have begun to elucidate the pathogenesis of this disease and to assess potential therapeutic agents. Among non-CBL genetic lesions, models with mutations of N-Ras, K-Ras, PTPN11 and others have been made [12–19]. Essentially all of these models show leukemogenesis when mice have attained adulthood (typically after 6–8 weeks of age). In the context of mutant CBL-driven myeloid malignancies, several models recapitulate the features of human disease to various extents [20–24]. Mice with a knock-in of a RF mutation in the CBL locus together with deletion of wildtype CBL gene develop delayed MPD, typically by 10 months of age [22]. We found that conditional deletion of CBL in HSCs on a CBL-B-null background using the mouse mammary tumor virus (MMTV) promoter-driven Cre, which was active in a small fraction of HSCs, led to a rapidly-progressive and lethal MPD by around 8 weeks of age [23]. Since an incidental CBL/CBL-B DKO in a minority of HSCs in these mice led to a model that recapitulated the aggressiveness of JMML but nonetheless with a delayed onset, we engineered a model in which CBL deletion was initiated in HSCs of CBL-B-null mice using VAV1-Cre, which is known to direct gene deletion during both fetal and adult hematopoiesis [25, 26]. We report here that the VAV1-Cre based CBL/CBL-B DKO mice are born at expected Mendelian ratios but succumb to a rapidly-progressive and fatal myeloproliferative disease beginning in the second week of life. This model recapitulates the early onset, aggressiveness, fatal disease and GM-CSF-hypersensitivity seen in JMML although without splenomegaly and with a massive accumulation of disease-initiating cells in the liver. The disease in this model is transplantable upon transfer of HSCs from the liver or bone marrow, highlighting the potential application of this model in mechanistic studies of the pathogenesis of JMML and in development and validation of therapeutic agents against JMML. This model should also be useful to investigate the roles of CBL proteins in regulating hematopoiesis during fetal and early neonatal life, which remain unknown.

## RESULTS

### VAV1-Cre mediated deletion of floxed-CBL on a CBL-B-null background leads to early neonatal lethality

**Figure 1:**
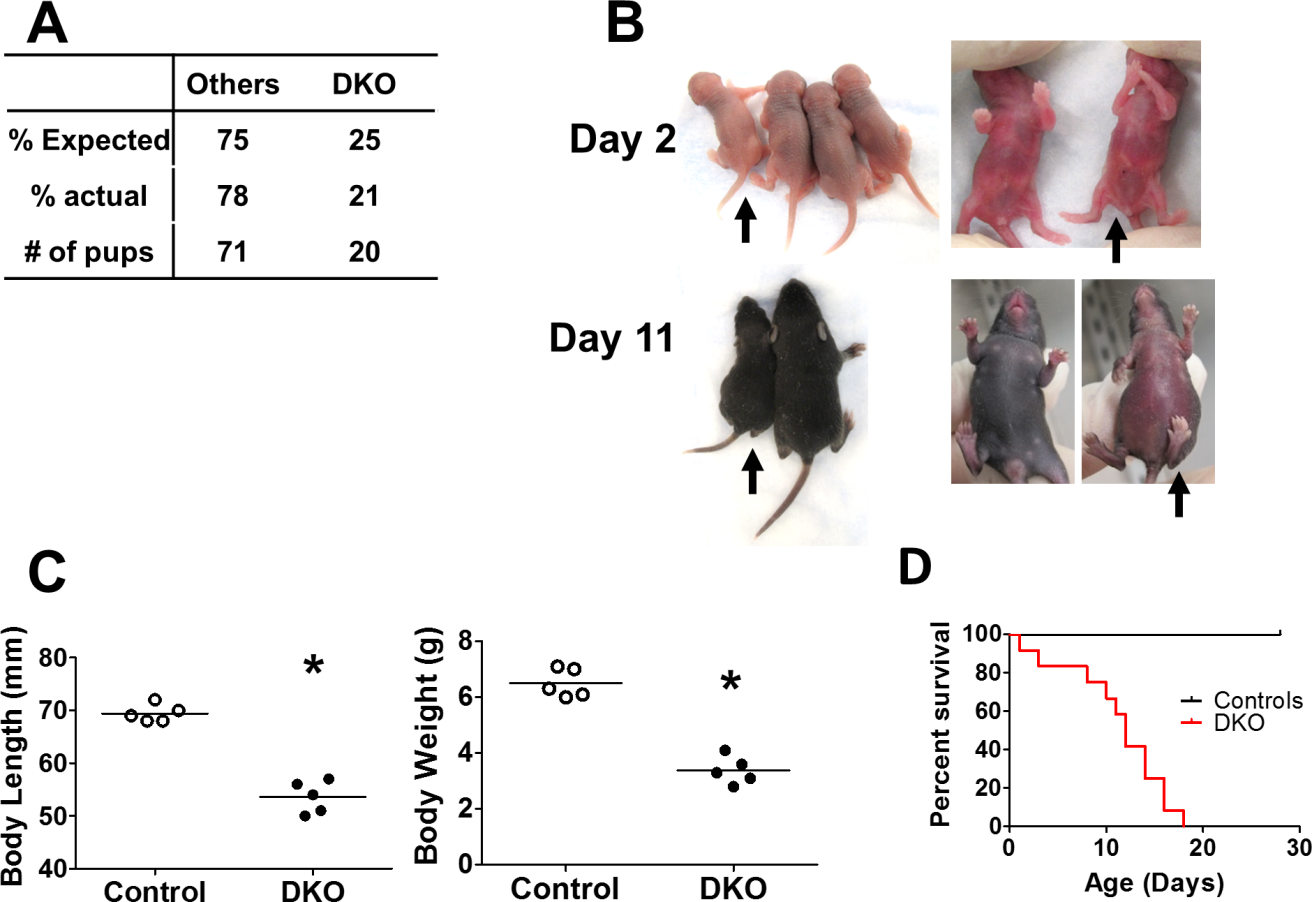
Embryonic deletion of CBL and CBL-B in hematopoietic system leads to rapid lethality (**A**) Expected and actual born ratio of DKO mice and controls (crossings of VAV ^Tg/0^ CBL^f/f^ CBL-B^+/–^ to CBL^f/f^ CBL-B^+/–^ mice). (**B**–**C**) DKO mice exhibit developmental disorder. (B) Representative photo. Black arrow indicates DKO pups. Others are littermate controls, which includes VAV ^Tg/0^ CBL^f/f^ CBL-B^+/–^, CBL^f/f^ CBL-B^–/–^ and CBL^f/f^ CBL-B^+/–^. (C) Quantification of body length and body weight at postnatal day 10. (**D**) Kaplan-Meyer survival curve. *n* = 12 for each group.

We established a new CBL/CBL-B conditional double knockout mouse model (DKO) in which floxed-CBL was conditionally-deleted with VAV1-Cre on a CBL-B null background. Among 91 pups born from crossings of VAV^Tg/0^ CBL^f/f^ CBL-B^+/−^ to CBL^f/f^ CBL-B^+/−^mice, 22% were CBL/CBL-B DKO, which is marginally lower than the expected Mendelian frequency of 25% (Figure 1A). Even though VAV1-Cre is known to be expressed beginning at day E11.5 [26], concurrent loss of CBL and CBL-B in the hematopoietic system using this Cre had only limited impact on embryonic development and was not pursued further.

Visual examination of 2-day old pups did not reveal any major defects in DKO pups compared to littermate controls, except for a slightly curled tip of the tail (Figure 1B). However, by day 10, DKO pups exhibited substantially delayed development, evidenced by the significantly reduced body length and body weight (Figure 1B and 1C). This was followed by rapid lethality, beginning around postnatal day 10 and all DKO pups succumbed by 20 days of age (Figure 1D).

### DKO mice exhibit a severe neonatal myeloproliferative disorder

**Figure 2:**
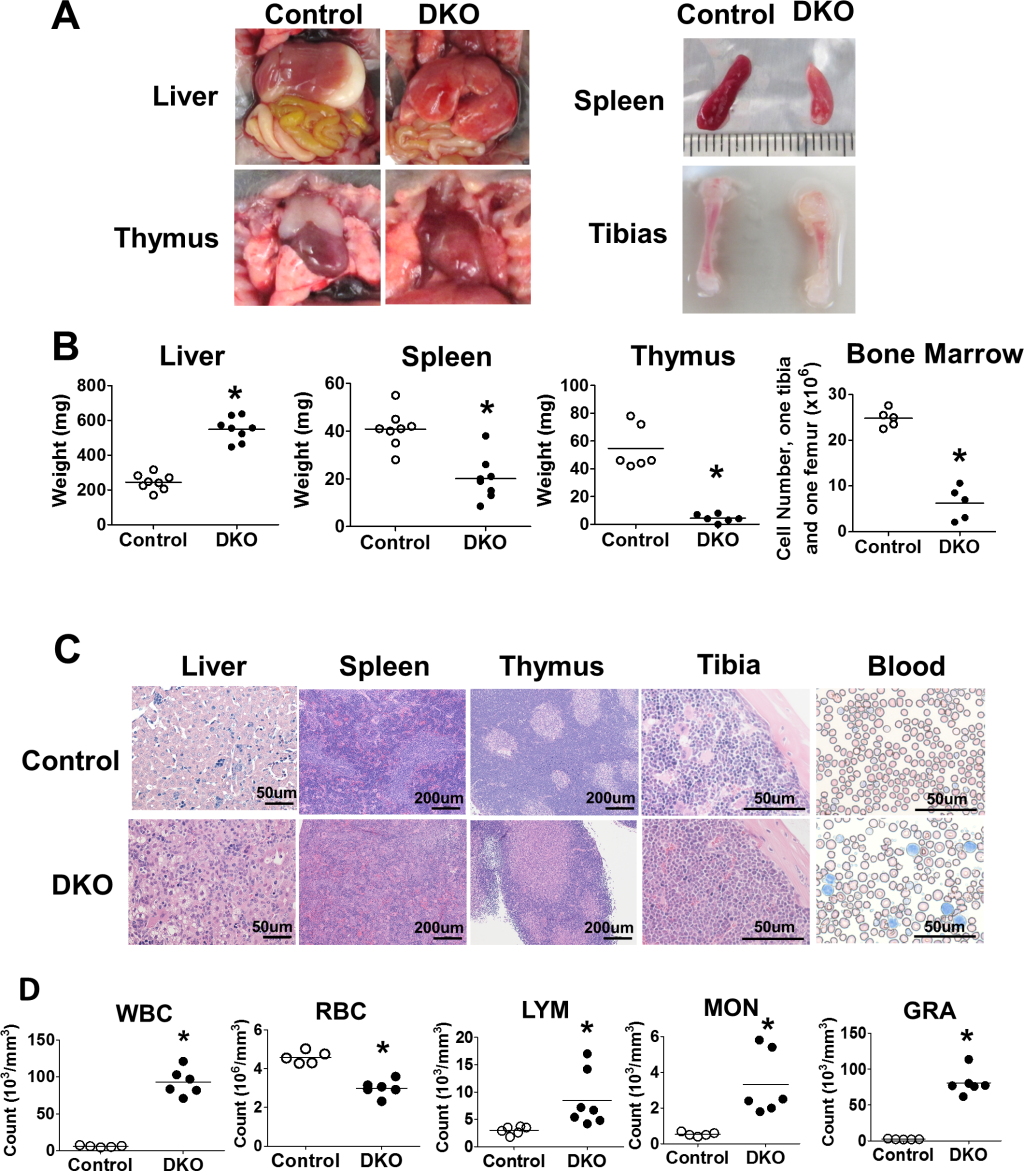
DKO mice exhibit severe neonatal leukemia (**A**–**C**) Anatomical analysis and H&E staining. (A) Photos representing liver, spleen, thymus and tibia. (B) Weight of liver, spleen, thymus and BM cell count. (C) H&E staining of liver, spleen, thymus, tibia and Wright-Giemsa staining of peripheral blood smear. (**D**) Peripheral blood cell count. Each dot represents an individual mouse. (*p < 0.05).

Analysis of the organs of DKO pups around day 10 or later revealed massive hepatomegaly as a notable abnormality (Figure 2A and 2B). H&E staining revealed extensive disruption of liver architecture and replacement with mononuclear infiltrates in VAV1-Cre DKO mice (Figure 2A–2C).Interestingly, in contrast to prominent splenomegaly that accompanies hepatomegaly in the MMTV-Cre-based CBL/CBL-B DKO model of MPD [23, 24], the splenic size was significantly reduced in VAV1-Cre-based DKO mice with paler spleens, which showed reduced red and white pulp cellularity upon H&E staining (Figure 2A–2C). Furthermore, thymus size was drastically reduced and barely visible, with dramatic loss of thymocytes in H&E sections (Figure 2A–2C). Long bones, as exemplified with tibia, of CBL/CBL-B DKO mice were shorter and paler, with marked reduction in the number of bone marrow cells. (Figure 2A–2C).

Given a dramatic hepatomegaly with mononuclear cell infiltration, we performed peripheral blood cell counts in littermate control vs. CBL/CBL-B DKO mice (Figure 2D). DKO mice exhibit dramatically increased total white blood cell (WBC) counts and reduced red blood cell (RBC) counts. Differential white blood cell counts further showed a significant increase in lymphocyte (LYM), monocyte (MON) and granulocyte (GRA) counts in DKO compared to control mice, with maximum increase observed in the GRA counts (Figure 2D). These observations supported the existence of a marked myeloproliferative disease together with expansion of lymphocytes in the periphery.

### Markedly elevated hepatic extramedullary hematopoiesis in VAV1-Cre-induced CBL/CBL-B DKO mice

**Figure 3:**
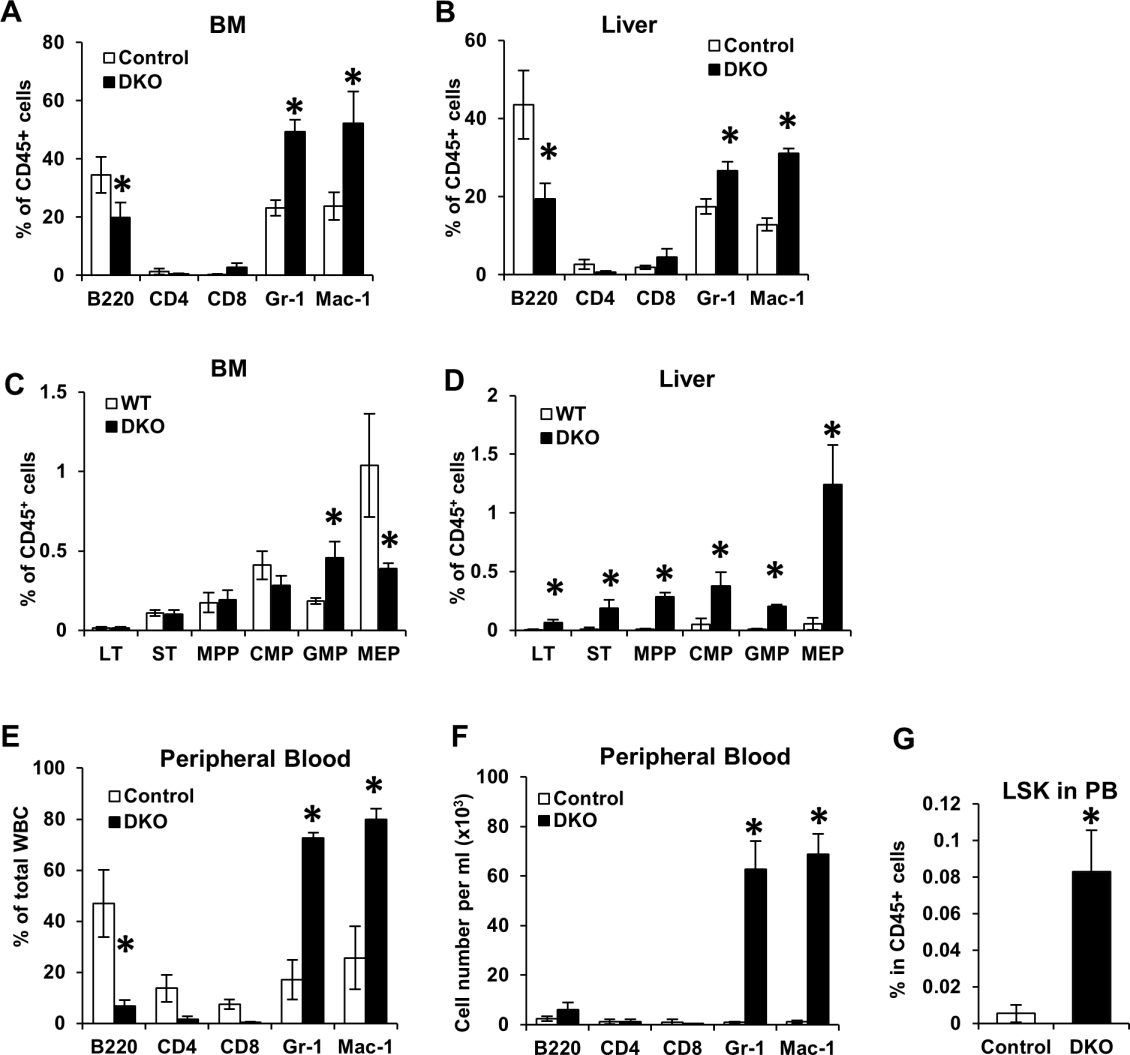
Loss of CBL and CBL-B leads to unique hematopoiesis features during neonatal development (**A**–**D**) BM and Liver were collected from WT control and DKO mice for FACS analysis. Percentage of mature cells (A–B) and stem/progenitors cells (C–D) were quantified from three independent experiments, shown as mean ± SD. Total percentage of CD45+ cells in A and B exceeds 100% due to overlap of Gr1+ and Mac1+ populations. (**E**–**G**) Peripheral blood were collected from WT control and DKO mice and analyzed by FACS. Percentage of mature cells (E), absolute number of mature cells (F) and percentage of LSK cells (G) were analyzed. Data is pooled from three independent experiments shown as mean ± SD.

In view of the marked hepatomegaly and hypoplasia of spleen and thymus that was accompanied by a dramatic peripheral blood leukocytosis in VAV1-Cre-induced CBL/CBL-B DKO mice, we next performed FACS analysis of selected tissues to further assess the nature of alterations in hematopoiesis. Since fetal hematopoiesis primarily takes place in the liver while adult hematopoiesis occurs in the BM, we primarily focused on neonatal BM and neonatal liver for further analyses. Consistent with the expansion of myeloid cells in the peripheral blood of DKO mice, these mice exhibited a dramatic increase in the number of Gr-1+ and Mac-1+ myeloid lineage cells in the liver and BM (Figure 3A and 3B). This was associated with a relative decrease in the proportion of B220+ B-lineage, which, based on the overall increase of WBC and LYM in the peripheral blood, was likely due to a disproportionate increase in myeloid cells (Figure 2D).

Multiple mouse models with perturbations in CBL-family genes, including the CBL KO, the CBL RF mutant knock-in and MMTV-Cre-induced CBL/CBL-B conditional DKO mice, exhibit an expansion of hematopoietic stem cells (HSCs) in the BM [21–24]. Given the marked hepatomegaly with relatively acellular BM in Vav1-Cre-induced CBL/CBL-B DKO mice, we analyzed the alteration of HSCs (Lineage−/SCA1+/c-KIT+ or LSK cells) in the BM and liver of these mice. Consistent with their hypo-cellular BM, the numbers of HSCs and common myeloid progenitors (CMP) in the BM of DKO mice were comparable to those of the controls. The DKO BM did however contain increased numbers of granulocyte-macrophage progenitors (GMP) and a reduced number of megakaryocyte-erythrocyte progenitors (MEP) compared to controls (Figure 3C). Notably, all hematopoietic stem and progenitors were found at dramatically increased numbers in the liver of CBL/CBL-B DKO mice (Figure 3D). Thus, consistent with the hypo-cellularity of the BM and increased mononuclear infiltration of the liver (Figure 2B), the VAV1-Cre-induced CBL/CBL-B DKO mice exhibited impaired BM hematopoiesis and enhanced hepatic extramedullary hematopoiesis, while the latter is apparently the primary source of peripheral leukocytosis.

During embryonic development, HSCs are released from the fetal liver and migrate into the BM and other organs such as the spleen and the thymus for adult hematopoiesis [27]. As neonatal liver was the only hematopoietic organ in VAV1-Cre-induced CBL/CBL-B DKO mice that showed an expansion of early hematopoietic cell lineages, we surmised that fetal HSCs in the DKO mice may not be able to leave the fetal/neonatal liver and enter the circulation. To address this, we assessed the presence of HSCs in the peripheral blood of DKO mice. Notably, in addition to significantly elevated percentages and total cell numbers of Gr-1+ and Mac1+ cells (Figure 3E and 3F), the peripheral blood of DKO mice also contained dramatically elevated levels of HSCs (LSK cells) (Figure 3G). This result suggests that DKO HSCs are able to be released from fetal/neonatal liver into circulation, making it unlikely that the dominant hepatic hematopoiesis is due to the inability of HSCs to exit from the liver.

### Increased colony-forming activity in cells from the liver and BM of VAV1-Cre-induced CBL/CBL-B DKO mice

**Figure 4:**
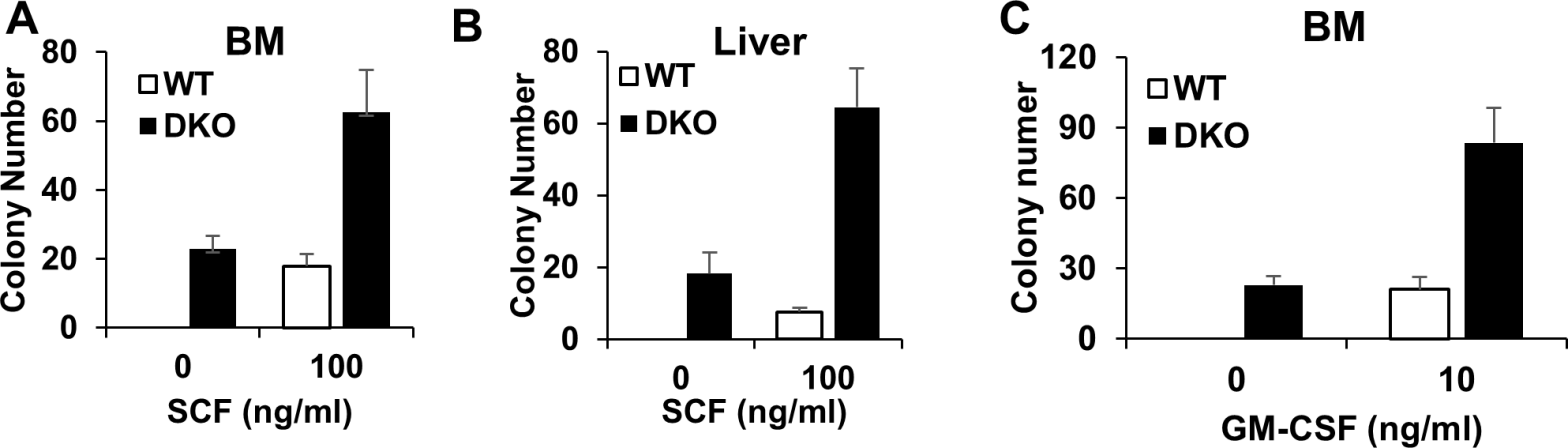
BM and liver in VAV DKO contain disease initiating cells (**A**–**B**) BM and Liver were collected from WT control and DKO mice for colony forming assay with or without SCF. (**C**) BM cells were collected from WT control and DKO mice for colony forming assay with or without GM-CSF. Data is pooled from three independent experiments, shown as mean ± SD.

Given our results that liver, but not BM, shows expansion of HSCs in the DKO mice (Figure 3B and 3C), we asked if this might represent a cell-intrinsic difference in the self-renewal and proliferation of HSCs in the liver vs. those in the BM, using a colony-forming assay. Equal numbers of BM and/or liver mononuclear cells isolated without any enzymatic dissociation from WT control or DKO mice were seeded in colony-forming cultures either in a cytokine-free medium or with stem cell factor (SCF). Interestingly, both BM and liver mononuclear cells isolated from the DKO mice exhibited cytokine-independent colony-forming ability, and a marked hyper-responsiveness to stimulation with SCF (Figure 4A and 4B). These results support the conclusion that DKO HSCs are hyper-proliferative and that the lack of expansion of HSCs in the BM does not reflect the presence of functionally-impaired HSCs.

*In vitro* hyper-responsiveness towards GM-CSF is an invariable feature of BM leukemic cells in JMML patients [5]. Given the recapitulation of key features of JMML, such as early neonatal MPD and rapid lethality, in VAV1-Cre CBL/CBL-B DKO mice we assessed if the BM cells in these mice exhibited GM-CSF hyper-responsiveness. As shown in Figure 4C, DKO BM cells indeed exhibit a significantly higher level of colony-forming ability when cultured in GM-CSF, further supporting that the VAV1-Cre-induced CBL/CBL-B DKO model recapitulates pathogenic features of JMML.

### Both liver and BM cells from VAV1-Cre-induced CBL/CBL-B DKO mice possess *in vivo* disease-initiating capability

**Figure 5:**
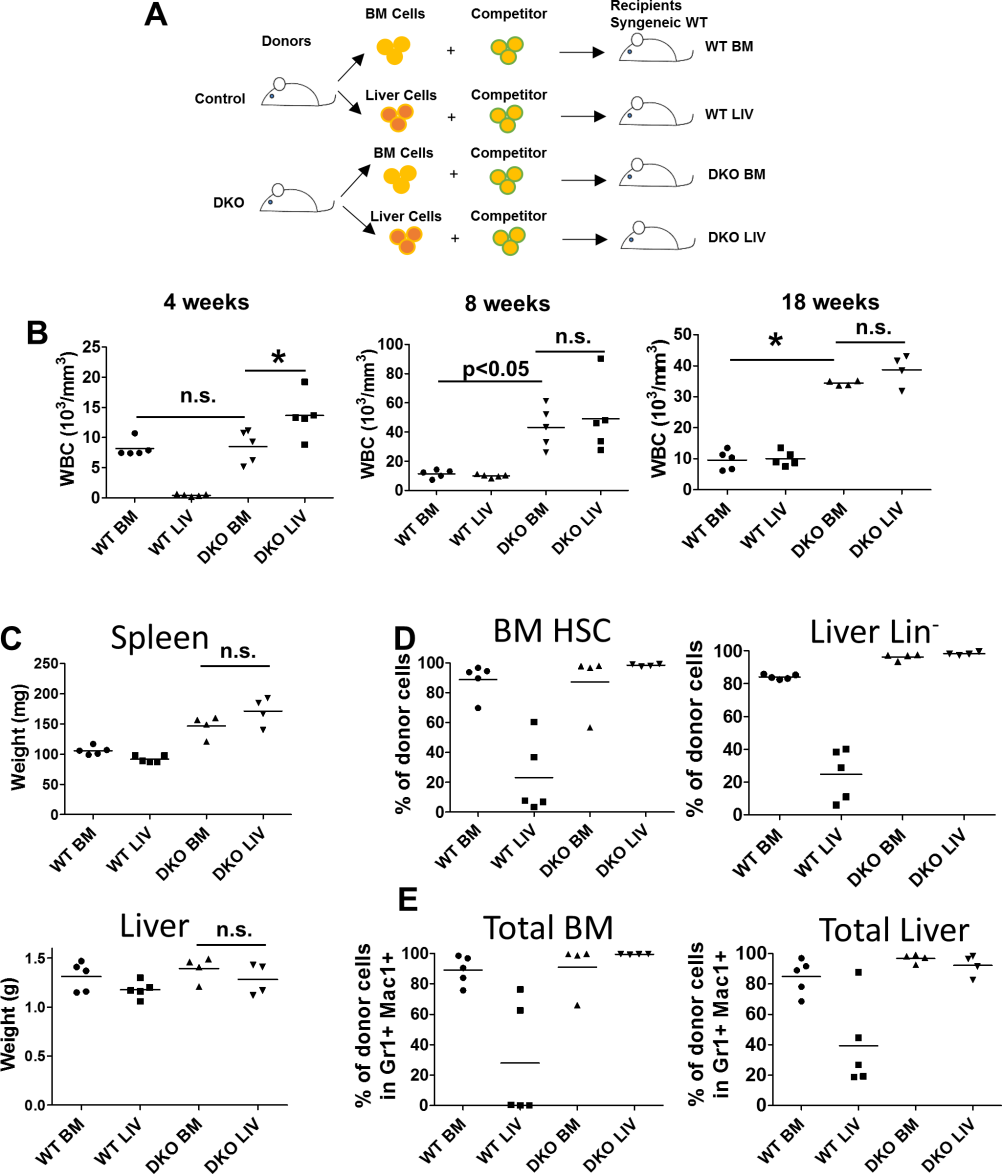
Both BM and liver cells from DKO mice were able to initiate leukemia (**A**) Experimental design. (**B**) Peripheral blood cell count was performed at indicated time point after transplantation of 2 M whole BM cells or liver cells together with 0.5 M competitor/helper cells. (**C**) Liver and Spleen were weighted at the end of the experiment. (**D**–**E**) BM and liver were collected and analyzed by FACS for percentage of stem/progenitor cells (D) or percentage total donor cells (E). Each dot represents an individual transplant recipient. (*p < 0.05, n.s.: not significant).

To further assess if the JMML-like MPD seen in VAV1-Cre-induced CBL/CBL-B DKO mice was transplantable *in vivo*, and to assess *in vivo* if the divergent expansion of HSCs in the liver vs. BM of DKO mice represents any cell-intrinsic differences, we performed transplantation experiments. For this purpose, neonatal liver or BM mononuclear cells from control or DKO mice were transplanted into lethally-irradiated recipients together with competitor BM cells (Figure 5A). Peripheral blood was analyzed at 4, 8 and 18 weeks after transplant (Figure 5B). Notably, DKO liver cells induced a rapid increase in the WBC count at 4 weeks after transplant compared to all other groups, consistent with the higher percentage of HSCs in liver mononuclear cells (Figure 3B). Leukocytosis was observed in mice transplanted with DKO liver or BM mononuclear cells beyond 8 weeks, while recipients of control BM or liver mononuclear cell transplants exhibited peripheral blood counts within the normal range, as expected. These results support the conclusion that HSCs in the liver as well as BM of VAV1-Cre-induced CBL/CBL-B DKO mice are intrinsically functional as MPD-initiating cells.

At 18 weeks after transplant, recipient mice were euthanized and their tissues were analyzed. In contrast to donor DKO mice, which exhibit significant hepatomegaly with a reduced splenic size, the recipient mice transplanted with either the DKO liver or the DKO BM mononuclear cells showed splenomegaly while the size of liver was comparable to that of control recipients (Figure 5C). These observations suggest that the hepatomegaly phenotype in donor DKO mice is unlikely a reflection of a leukemic cell-intrinsic defect that results in retention in liver.

We also analyzed the levels of donor cell-derived HSCs and myeloid cells in the recipients’ BM and liver. As expected from the low frequency of HSCs in normal liver, mice transplanted with WT liver mononuclear cells exhibited significantly lower levels of HSCs in the BM and liver compared to those receiving the WT BM mononuclear cells (Figure 5D). In contrast, recipients transplanted with DKO BM or liver mononuclear cells exhibited equivalent reconstitution of BM LSK cells and liver Lin^−^ cells, suggesting an intact migration ability of DKO BM and liver derived HSCs (Figure 5D). Similar results were observed through analysis of myeloid cells, with DKO BM or liver mononuclear cell transplants resulting in equivalent mature myeloid cell expansions in the liver and BM of recipient mice (Figure 5E). Together, these data support the conclusion that MPD-initiating cells are present in both liver and BM of VAV1-Cre-induced CBL/CBL-B DKO mice and that the relative lack of expansion of such cells in the BM and spleen of donor DKO mice may reflect a non-cell autonomous defect in a niche component in VAV1-Cre-induced CBL/CBL-B DKO mice.

## DISCUSSION

CBL-family proteins function as essential negative regulatory elements of downstream signaling linked to activation of PTKs. Consistent with the expression of CBL and CBL-B in the hematopoietic system, mutations in CBL, and rarely CBL-B, are associated with certain forms of myeloid leukemia, with as many as 15% cases of JMML due to mutations in CBL. Given the neonatal onset and aggressive course of JMML, limited therapeutic armamentarium and only about 50% 5-year remission with bone marrow transplant, experimental models that closely replicate this neonatal malignancy are likely to open new approaches to understand JMML pathogenesis and identify new therapeutic approaches [10]. Based on our previous observations that incidental conditional CBL deletion in a small fraction of HSCs of CBL-B-null mice, using MMTV-Cre, led to CBL/CBL-B double knockout model with a rapidly-fatal MPD around 8 weeks of age [23], we hypothesized that the prenatal and early neonatal deletion of CBL on a CBL-B-null background using VAV1-Cre could result in a more robust and early CBL/CBL-B DKO in HSCs and produce an early neonatal model of MPD, more closely representative of JMML-associated pathogenic mechanisms. Here, we establish such a model and demonstrate that hematopoietic-specific conditional deletion of CBL with VAV1-Cre, on a CBL-B-null background, yields a model in which a severe MPD starts in infancy and the disease is rapidly-fatal.

JMML occurs in children less than four years of age and is often diagnosed in infants [5, 7]. The most common molecular drivers of JMML are mutations in RAS signaling pathway regulators, including PTPN11, N-Ras, K-Ras, NF-1 and CBL [5, 7, 11]. While a number of animal models in which the human JMML-associated mutations are modeled in mice, they invariably produce a disease with an adult onset (at or after 8 weeks of age) [12–19]. The early onset MPD in the VAV1-Cre induced CBL/CBL-B DKO mice (Figure 1B) therefore provides a novel mouse model relevant to the pathogenesis of human JMML. Additionally, the VAV1-Cre induced DKO mice are born without overt developmental abnormalities and at nearly the expected Mendelian ratios (Figure 1A). These mice exhibit a dramatically quick disease progression that is invariably fatal (Figure 1D), further mimicking the clinical course of JMML. Therefore, the new mouse model could provide a new research tool to understand and target JMML that is associated with CBL mutations. However, since the molecular drivers in JMML appears to involve a common aberration of RAS signaling, the present model might provide a model more broadly relevant to pathogenic mechanisms that drive JMML pathology in general.

At the pathological level, a number of features of the hematopoietic disease in VAV1-Cre-induced CBL/CBL-B DKO mice resemble those seen in JMML, as well as in previous models with CBL/CBL-B deletion or CBL RF mutation [22–24]. These include a prominent hepatomegaly (Figure 2A and 2B) with peripheral leukocytosis that is heavily skewed towards myeloid (monocyte, neutrophil) lineages but with some lymphocytosis as well (Figure 2D), and hypersensitivity of BM cells to cytokines, including GM-CSF (Figure 4C), a hallmark of JMML [28]. Notably, however, the VAV1-Cre-induced CBL/CBL-B DKO mice exhibit smaller and hypo-cellular spleens (Figure 2B), a hypo-cellular bone marrow without any evidence of HSC compartment expansion (Figure 2B and Figure 3C) evident in previous models of CBL mutation-driven MPD [22–24]. These mice also exhibit a poorly-developed thymus (Figure 2B), similar to the thymic abnormality in CBL RING finger mutant mice [29]. Nonetheless, FACS analysis revealed the presence of HSCs in the BM as well as increased myeloid progenitors and mature myeloid cell populations, suggesting that a certain degree of abnormal hematopoiesis does occur in the bone marrow. The liver, on the other hand, harbored a dramatically increased HSC load and the hepatic architecture is markedly abnormal with large mononuclear infiltrates (Figure 2C and Figure 3D). Thus, the current model shows certain unique features that are not representative of JMML pathology and will need to be borne in mind while using the new mouse model for JMML-associated studies. Despite these deviant features in the DKO mice, both the hepatic and bone marrow cells could recapitulate MPD upon transplant and the recipients showed a prominent splenomegaly (Figure 5A–5E). Collectively, these findings support the likelihood that early embryonic and neonatal CBL/CBL-B DKO using VAV1-Cre creates a non-cell autonomous defect that restricts the extramedullary hematopoiesis to the liver while reducing bone marrow and splenic hematopoiesis.

Interestingly, compared to the rapid lethality of the donor mice (Figure 1D), the disease progression in the transplanted mice was slower (Figure 5B). Although severe MPD was seen by the time of the analysis (Figure 5B, 5C), the DKO BM/liver cell transplants did not lead to rapid lethality. The early fetal deletion of CBL and CBL-B deletion in all HSCs in the model described here may promote a more rapid loss of the hematopoietic reconstitution potential than we noted in our previous study of MMTV-Cre induced CBL deletion on a CBL-B-null background, which leads to CBL deletion in small fraction of HSCs [24]. Alternatively, this observation might reflect a difference between fetal and adult hematopoiesis, as the transition of fetal to adult hematopoiesis is known to involve significant cell-intrinsic changes in HSCs as well as in their microenvironment [27]. Notably, a reduction in the aggressiveness of JMML disease in patients with CBL mutations has been reported with age in some clinical reports and spontaneous remissions can occur [5, 30, 31]. However, more in-depth analyses of the fetal and neonatal HSCs in the current DKO model in comparison with those in our previous model will be needed to more precisely address these issues.

The VAV1-Cre transgene is known to be hematopoietic lineage-restricted [25, 26]. However, BM, and potentially other lymphoid organ, niches are known to contain cells of hematopoietic origin (such as osteoclasts and macrophages in the BM) [32, 33]. The increased numbers of lineage-positive DKO macrophage in the BM (Figure 3A) could possibly contribute to disruption of the BM niche. In addition, cytokines secreted by hematopoietic cells are known to regulate hematopoiesis and aberrations of cytokine production by DKO cells could be a contributing factor [32, 33]. It also remains possible that loss of CBL/CBL-B causes defective migration of fetal HSCs into BM and to other lymphoid organs, with MPD-initiating cells in the BM entering secondarily due to their increased numbers in the peripheral circulation (Figure 3G). Further investigations will be needed to determine if the liver-skewed extramedullary hematopoiesis in the VAV1-Cre CBL/CBL-B DKO model represents a cell-intrinsic or a non-cell autonomous defect (e.g., components of niche or secreted factors).

It is well accepted that HSCs reside in the fetal liver during late embryonic development and then migrate to the BM, spleen and other extramedullary organs. Notably, VAV1-Cre is known to be expressed at around E11.5, when HSCs reside primarily in the fetal liver. Thus, the dominant hepatomegaly in our model may reflect the initial site of expansion of DKO HSCs in the fetal liver. Although we have not examined the fetal liver in our model, the fact that these mice are born without significant embryonic lethality suggests that the major thrust of the disease development is post-natal. This is consistent with our understanding of hematopoiesis, with a majority of fetal HSCs being fast-cycling cells [34]. As we have shown previously [24], a major impact of combined CBL/CBL-B deletion in HSCs in the MMTV-Cre-induced model was a significant shift of quiescent to cycling HSCs. As such, the impact of CBL/CBL-B deficiency during fetal life may not be immediately apparent. However, during transition of fetal to adult hematopoiesis, HSCs rapidly attain quiescence [27, 33, 34]. It is likely that lack of CBL/CBL-B negates this process, further expanding the pool of HSCs and induces an early neonatal MPD, while also rapidly depleting their reconstitution potential. Further studies to experimentally determine if CBL-family proteins indeed are involved in promoting the entry of fetal-cycling HSCs into quiescent HSC pool during late fetal and early neonatal life will be of great interest.

In conclusion, we report the establishment of hematopoietic system-specific CBL/CBL-B DKO mice and provide support that this represents a novel animal model that recapitulate key features of the infancy onset disease observed in JMML patients. To our knowledge, this is the first mouse model with severe and lethal MPD occurring at infancy, and should provide a useful tool for future mechanistic and therapeutic development studies of JMML as well as to understand the role of CBL-family proteins in fetal and early neonatal hematopoiesis.

## MATERIALS AND METHODS

### Animals

VAV1-Cre were from the Jackson Laboratory and were crossed with CBL-flox/flox CBL-B-null mice [23], maintained on a C57/Bl6 background [24]. DKO mice were generated by crossings of VAV^Tg/0^ CBL^f/f^ CBL-B^+/−^ to CBL^f/f^ CBL-B^+/−^ mice. B6.SJL-Ptprca Pepcb/BoyJ mice (The Jackson Laboratory) were used as transplant recipients. F1 generation of crosses between C57/BL6 and B6.SJL-Ptprca Pepcb/BoyJ mice were used to provide competitor cells for BM transplantation. Mice were housed under specific pathogen-free conditions and sacrificed for analysis at the indicated times. Mouse studies were reviewed and approved by the Institutional Animal Care and Use Committee.

### Reagents and antibodies

The murine recombinant cytokines SCF, TPO, IL-3, IL-6 and FLT3L were obtained from PeproTech MethoCult M3234 Methylcellulose medium was obtained from Stem Cell Technologies. The following antibodies for flow cytometry were obtained from eBioscience: CD45.1 (A20); CD45.2 (104); Sca-1 (D7); c-Kit (2B8); CD16 (93). CD34 (RAM34) and FLT3 (A2F10.1) antibodies were obtained from BD biosciences. Fetal bovine serum (FBS) was from Hyclone. Biochemicals were from Sigma or Life Technologies unless indicated.

### BM preparation and FACS analysis

Whole bone marrow cell suspensions were prepared from femurs and tibiae. For stem and progenitor cell analysis and sorting, mature hematopoietic cells (lineage-positive cells) were labeled with antibodies against CD5, B220, CD11b, Gr-1, and 7–4 (mouse lineage depletion kit; Miltenyi Biotechnology) and magnetically depleted using the autoMACS (Miltenyi Biotechnology). Lineage-negative cells were then stained with antibodies followed by cell analysis or sorting. Flow cytometry was performed on a BD LSRII or Aria II at the UNMC Flow Cytometry Research Facility. Data were analyzed using FlowJo software (Tree Star). Cell populations were defined as follows [32, 33, 35] LT-HSC: CD34^−^ FLT3^−^ Lin^−^ Sca-1^+^ c-Kit^+^; ST-HSC: CD34^+^ FLT3^−^ Lin^−^ Sca-1^+^ c-Kit^+^; MPP: CD34^+^ FLT3^+^ Lin^−^ Sca-1^+^ c-Kit^+^; LSK: Lin^−^ Sca-1^+^ c-Kit^+^; CMP: CD16^−^ CD34^+^ Lin^−^ Sca-1^−^ c-Kit^+^; GMP: CD16^+^ CD34^+^ Lin^−^ Sca-1^−^ c-Kit^+^; MEP: CD16^−^ CD34^−^ Lin^−^ Sca-1^−^ c-Kit^+^; CLP: IL-7R^+^ FLT3^+^ Lin^−^ Sca-1^low^ c-Kit^low^.

### Colony-forming assays

20,000 of cells were cultured in MethoCult M3234 (StemCell Techologies) with indicated cytokines (all from PeproTech) for 7 days, followed by colony counting.

### Statistics

Unpaired Student's *t* test on Prism was used to calculate the *p* values. *p < 0.05.
